# Reliability of radiographic measurements of knee motion following knee arthroplasty for use in a virtual knee clinic

**DOI:** 10.1308/003588412X13373405385575

**Published:** 2012-10

**Authors:** A Phillips, A Goubran, S Naim, D Searle, V Mandalia, A Toms

**Affiliations:** Royal Devon and Exeter NHS Foundation Trust,UK

**Keywords:** Knee, Outpatient, Radiograph

## Abstract

**INTRODUCTION:**

We sought to validate radiographic measurements of range of motion of the knee after arthroplasty as part of a new system of virtual clinics.

**METHODS:**

The range of motion of 52 knees in 45 patients was obtained by 2 clinicians using standardised techniques and goniometers. Inter-rater reliability and intraclass correlation coefficients (ICCs) were calculated. Radiographs of these patients’ knees in full active flexion and extension were also used to calculate intra and inter-rater reliability compared with clinical measurements using four different methods for plotting angles on the radiographs.

**RESULTS:**

The ICC for inter-rater reliability using the goniometer was very high. The ICC was 0.91 in extension and 0.85 in flexion while repeatability was 8.49° (-8.03–8.99°) in extension and 5.23° (-4.54–5.74°) in flexion. The best ICC for radiographic measurement in extension was 0.86, indicating ‘near perfect’ agreement, and repeatability was 5.43° (-4.04–6.12°). The best ICC in flexion was 0.95 and repeatability was 5.82° (-3.38–6.55°). The ICC for intrarater reliability was 0.98 for extension and 0.99 for flexion on radiographic measurements.

**CONCLUSIONS:**

Validating the use of radiographs to reliably measure range of motion following knee arthroplasty has allowed us to set up a ‘virtual knee clinic’. Combining validated questionnaires and radiographic measurement of range of motion, we aim to maintain high quality patient surveillance following knee arthroplasty, reduce our ratio for new to follow-up patients in line with Department of Health guidelines and improve patient satisfaction through reduced travel to hospital outpatients.

Total knee replacement (TKR) is a very common operation in the UK. Almost 82,000 knee replacements were carried out in England and Wales during 2010 and the number is rising each year.^1^ Each patient requires multiple follow-up outpatient appointments but increasing pressure on the primary care trust to fulfil its statutory obligation to increase efficiency and rationalise commissioning has led to the targeting of these outpatient episodes as a potential source of cost reduction. This has been highlighted in the Deparment of Health’s publication *National Standards, Local Action*.^2^

Despite the current economic pressure, the British Orthopaedic Association would advocate that resources ‘must be made available for prolonged follow-up and data from each Trust should be available and obtainable in a common format for regional and national audits’.^3^ This is required not just for patient surveillance but for clinical governance concerning the longevity and outcome using prosthetic implants. Long-term follow-up results are also used for research purposes.

Follow-up for TKR has been recommended to continue for a minimum of ten years^3^ with clinical examination and radiographic analysis being the gold standard. This is acknowledged as being difficult logistically so current recommendations are for radiological surveillance for failure, not function, at five-yearly intervals from five years post-operatively. This is due to the fact that the risk of failure and need for revision rises slowly at first but increases greatly after ten years. Failure of the prosthesis can often be silent. It is therefore of paramount importance for a regular review to occur to prevent asymptomatic patients having catastrophic complications that could have been picked up earlier. Furthermore, intervention has to be timed appropriately before massive bone destruction occurs,^3^ which may compromise revision surgery.

We are working with the primary care trust in Devon to set up ‘virtual clinics’ so that patients have radiographs of their TKR locally and fill in postal or internet-based questionnaires instead of attending the outpatient clinic. This would only be applicable to patients who are not experiencing problems and have returned for one post-operative visit at six weeks already. We believe that adequate follow-up outcome measures for these virtual clinics include the Western Ontario and McMaster Universities Arthritis Index,^4^ the Oxford knee score,^5^ the patients’ range of motion of the knee (measured on a radiograph) and a weight bearing radiograph to assess for loosening and alignment.

Anatomical landmarks to plot an angle on a lateral radiograph of a patient’s knee have not been validated in the literature despite radiographs having been used as a gold standard.^6,7^ We sought to establish these landmarks to allow us to plot a patient’s range of motion ‘virtually’ from a radiograph instead of face-to-face clinical examination.

Radiographs have been used to assess range of motion in the spine^8–10^ but to our knowledge no research has been published to assess range of motion in the knee using radiographs. Brosseau *et al* analysed intra and intertester reliability of two different goniometers and compared their findings with a radiograph taken in flexion and extension.^7^ The radiograph was considered the gold standard but landmarks on the tibia and fibula were not tested and errors may have been introduced by this method. They found that intertester reliability was high in flexion but lower in extension. We aimed to establish anatomical landmarks on radiographs in flexion and extension that reproduce goniometric measurements reliably. As part of ongoing service evaluation and enhancement we proposed:

1. We can measure patients’ range of motion reliably by using lateral radiographs in full active flexion and extension instead of by clinical measurements.
2. Questionnaires can be posted to patients to fill in at home and sent back to us.

These measures would negate the need for face-to-face consultation unless a complication arises or a significant drop in score is noted.

## Methods

### Patients

A total of 43 patients requiring radiographs of their knee at their next visit were identified on the Exeter Knee Reconstruction Unit outpatient arthroplasty database at the Princess Elizabeth Orthopaedic Centre at Royal Devon and Exeter Hospital (RD&E). Overall, 52 examinations in 50 knees were included in the study. Two patients were examined at six months and again at one year post-operatively. Six patients had bilateral TKRs.

Inclusion criteria were that the patients would require routine post-operative radiographs at their next appointment following knee replacement. Patients were either returning for a check radiograph at six months or routine radiographic surveillance at one, two, five or ten years after surgery. No patients were excluded.

Patients were invited to attend study clinics for evaluation. There were 22 women and 21 men with an average age of 73 years (range: 45–92 years; standard deviation: 9.2). Overall, 48 knees in 40 patients received a Scorpio® NRG total knee replacement (Stryker, Allendale, NJ, US), 1 patient had a Triathlon partial knee replacement (Stryker) and 2 patients had a Journey™ Deuce™ bicompartmental knee replacement (Smith & Nephew, Memphis, TN, US) including a patient who had two examinations.

### Radiographs

Standardising the position of the limb when taking measurements increases the reliability coefficient.^11^ A protocol for positioning during radiographs (and examination) was therefore developed:

1. Weight bearing anteroposterior radiograph
2. Lateral radiograph with knee in full active flexion; patient supine, knee pointing vertically with foot flat on the radiography table pointing directly forwards
3. Active ‘heel hang’: lateral radiograph in full active extension with the patient supine. A roll was placed under the Achilles tendon and the patient was instructed to push the knee towards the table with the foot pointing vertically.

The radiographers used the largest frame available (35cm × 43cm) to fit as much of the thigh and leg on the radiograph as possible to assist with plotting measurements.

### Testers

All the testers were full-time members of staff at the RD&E. Every patient was reviewed by a specialist registrar in trauma and orthopaedics and by another tester. The second tester was either a surgical care practitioner or senior radiographer. All testers were trained to use the goniometer in the same way and to identify the anatomical landmarks.

### Anatomical landmarks

Consistent anatomical landmarks corresponding to the joint lines of the hip, knee and ankle were established. The centre of the hip joint was designated as being at the proximal apex of the greater trochanter, the centre of the knee joint corresponded to a point on the joint line approximately 1.5cm proximal and anterior to the tip of the fibula,^12^ and the centre of the ankle corresponded to a point on the anterior border of the distal fibula.

### Instrumentation

**Figure 1:**
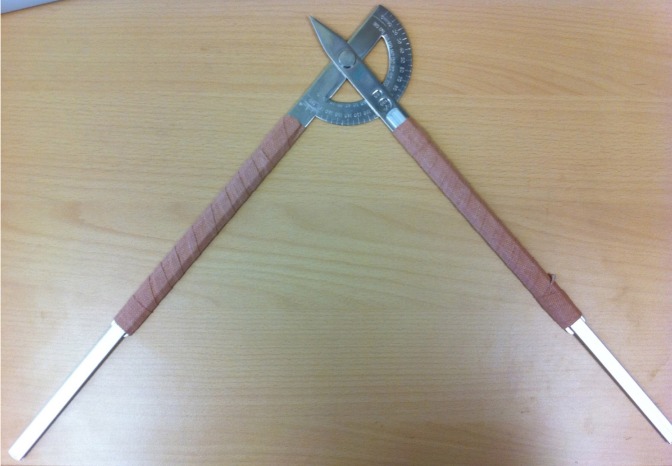
Extended goniometer

To improve accuracy, two universal goniometers were extended by means of straight metal rods to allow positioning on the centres of the three joints simultaneously (Fig 1). Training in identifying the landmarks and the use of the goniometers was undertaken for all testers.

### Procedure

Patients attending the study clinics would first attend the radiography department. A weight bearing anteroposterior radiograph was obtained. The patient was then instructed to lie flat on the radiography table. The knee was positioned for the radiograph in full active flexion and then active ‘heel hang’. Measurements were taken by the first tester immediately before each lateral radiograph. Patients were seen in the clinic room for a second measurement by the second tester. Routine follow-up discussion with the patient was also conducted and the radiographs were inspected for alignment and loosening.

### Radiography plots

**Figure 2:**
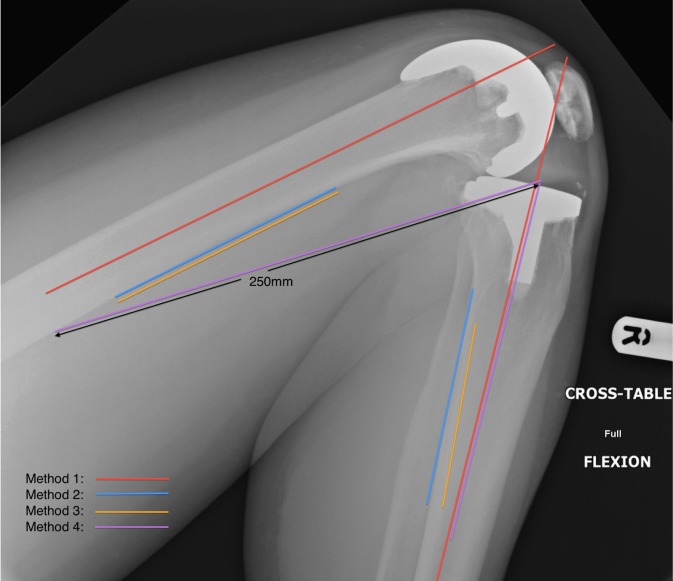
Flexion plots

**Figure 3:**
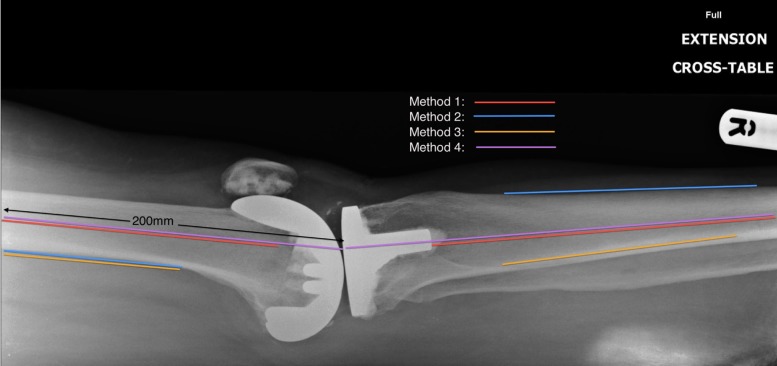
Extension plots

Four different plots were made for each radiograph using different anatomical landmarks on a web-based picture archiving and communication system (WebPACS). The angle was measured at the intersection of the femoral and tibial lines in flexion (Fig 2) and extension (Fig 3):

### Method 1

Flexion and extension:

Intramedullary lines drawn along long axes of the femur and tibia^7^

### Method 2

Flexion:

Tibia: posterior border of tibia distal to metaphyseal flare

Femur: posterior border of femur proximal to metaphyseal flare

Extension:

Tibia: anterior border of tibia distal to tibial tuberosity

Femur: posterior border of femur proximal to metaphyseal flare

### Method 3

Flexion and extension:

Fibula: anterior border just distal to the head of the fibula

Femur: posterior border of femur proximal to metaphyseal flare

### Method 4

Flexion:

Tibia: intramedullary line along long axis of tibia

Femur: 250mm (using WebPACS ruler) line connecting the posterior femur to a point on the joint line at the intersection of the tibial plot

Extension:

Tibia: intramedullary line along long axis of tibia

Femur: 200mm (using WebPACS ruler) line connecting the middle of the intramedullary cavity of the femur to a point on the joint line at the intersection of the tibial plot

Plots were made on each lateral radiograph using the four methods and the measurements documented. Each plot was performed twice on separate occasions to establish intratester reliability and a second independent clinician performed each plot to establish intertester reliability. Statistical analysis was performed to ascertain the intraclass correlation coefficient and repeatability for each plot. Bland–Altman plots were created to illustrate the differences in measurements between radiographs and goniometric measurements for each method.

### Data analysis

The statistics were calculated using StatsDirect v2.6.6 (StatsDirect, Altrincham, UK). After the data had been collected, the mean and standard deviations of the goniometer and radiographic measurements (in degrees) were calculated. Agreement and repeatability were explored using Bland–Altman analysis to compare repeated measures of range of motion within and between subjects. The intraclass correlation coefficient (ICC) (one-way random effects) was calculated to compare the goniometric and radiographic measurements. The ICC and its 95% confidence interval were also used to determine the interobserver reliability.^13^ Values less than 0.20 indicated ‘slight’ agreement, 0.21–0.40 indicated ‘fair’ agreement, 0.41–0.60 indicated ‘moderate’ agreement, 0.61–0.80 indicated ‘substantial’ agreement and values of 0.81 and greater indicated ‘near perfect’ agreement.^14^ The repeatability statistic is half of a 95% confidence interval, which is calculated from the standard deviation of all the differences between paired measurements. This indicates that a measurement taken on the radiograph may vary between the value of the repeatability statistic (in degrees).

## Results

ICCs and repeatability statistics were calculated, namely intertester ICC of goniometric measurements (Table 1), accuracy of radiographic to goniometric measurements (Table 2), intratester ICC of radiographic measurements (Table 3) and intertester ICC of radiographic measurements (Table 4).

**Table 1:** Intertester intraclass correlation coefficients and repeatability for goniometric measurements

Test	ICC	Repeatability	95% CI
Extension	0.91	8.49°	-8.03–8.99°
Flexion	0.85	5.23°	-4.54–5.74°

ICC = intraclass correlation coefficient; CI = confidence interval

**Table 2:** Intraclass correlation coefficients comparing radiographic and goniometric measurements

Method	Test	ICC	Repeatability	95% CI
1	Flexion Extension	0.83 0.76	11.46° 8.53°	-12.61–5.05° -9.43–7.24°
2	Flexion Extension	0.86 0.70	10.28° 9.49°	-11.63–7.27° -10.61–4.96°
3	Flexion Extension	0.81 0.73	12.60° 8.66°	-13.92–5.75° -8.48–8.98°
4	Flexion Extension	0.95 0.86	5.82° 5.43°	-3.38–6.55° -4.04–6.12°

ICC = intraclass correlation coefficient; CI = confidence interval

**Table 3:** Intratester intraclass correlation coefficients of radiographic measurements

Method	Test	ICC	Repeatability	95% CI
1	Flexion Extension	0.99 0.99	1.53° 1.06°	-1.48–1.58° -1.09–1.05°
2	Flexion Extension	0.99 0.99	1.15° 0.81°	-1.27–0.99° -0.85–0.78°
3	Flexion Extension	0.99 0.99	1.36° 1.23°	-1.48–1.21° -1.26–1.23°
4	Flexion Extension	0.99 0.99	1.65° 1.25°	-1.61–1.73° -1.34–1.15°

ICC = intraclass correlation coefficient; CI = confidence interval

**Table 4:** Intertester intraclass correlation coefficients of radiographic measurements

Method	Test	ICC	Repeatability	95% CI
1	Flexion Extension	0.95 0.91	6.33° 6.28°	-6.88–5.69° -6.74–5.81°
2	Flexion Extension	0.99 0.95	1.97° 4.64°	-1.43–2.24° -4.96–4.34°
3	Flexion Extension	0.99 0.90	2.09° 6.60°	-1.40–2.36° -7.08–6.14°
4	Flexion Extension	0.95 0.98	5.41° 1.92°	-6.01–4.48° -1.71–2.09°

ICC = intraclass correlation coefficient; CI = confidence interval

Agreement between the two testers for goniometric measurements was excellent (Table 1) as described previously in the literature.^6,7,15^

Method 4 had the highest ICC, lowest repeatability and narrowest confidence intervals, indicating that this method will produce less error when plotting range of motion on the radiograph (Table 2). This would be intuitively more accurate as this represents goniometric landmarks at the joint line most closely and therefore the true axis. Adjusting the femoral landmark on the radiographs between extension and flexion (from 200mm to 250mm respectively) was necessary to reduce the repeatability and increase the ICC.

Intratester reliability for radiographic measurements was ‘near perfect’ for all methods (Table 3). It was not possible to examine intratester reliability for goniometric measurements due to the inconvenience to the patients of bringing them back to the clinic for a second measurement on a separate occasion.

Intertester reliability of radiographic measurements showed a very high degree of correlation for all measurements (Table 4) but method 4 obtained much higher ICC and narrower confidence intervals in extension than previously documented in the literature.

**Figure 4:**
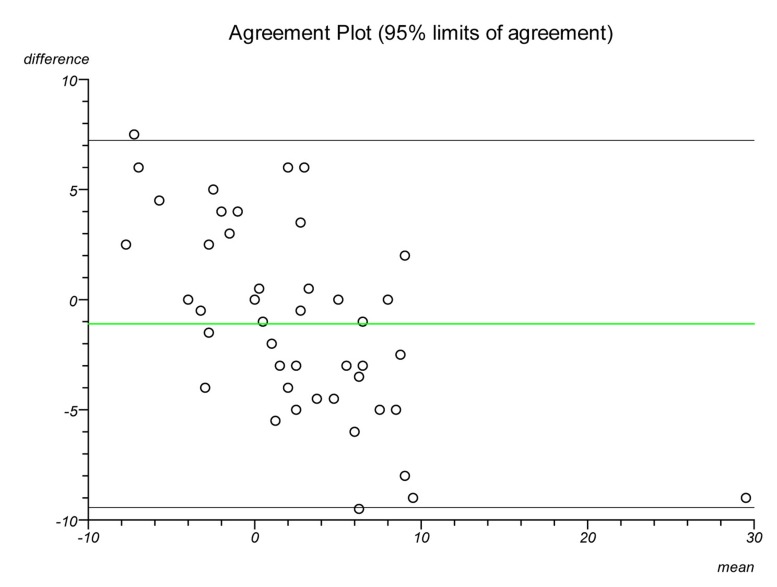
Radiographic plots using method 1 versus goniometer (extension)

**Figure 5:**
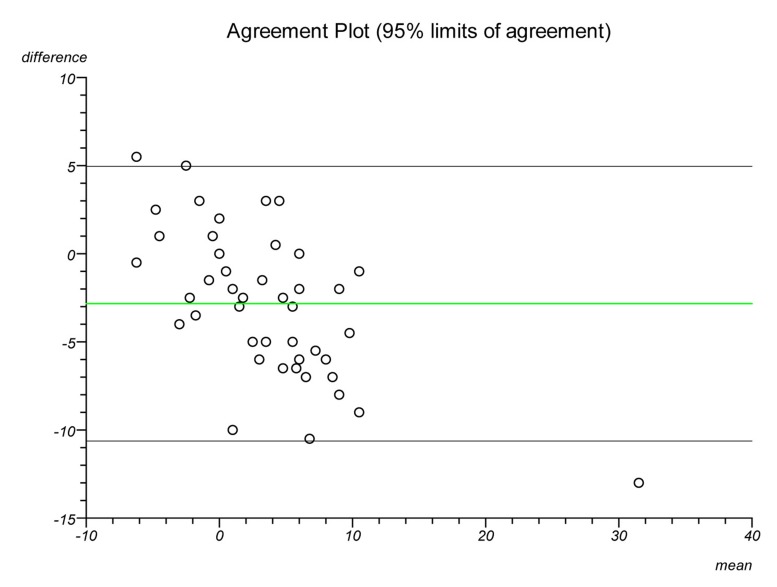
Radiographic plots using method 2 versus goniometer (extension)

**Figure 6:**
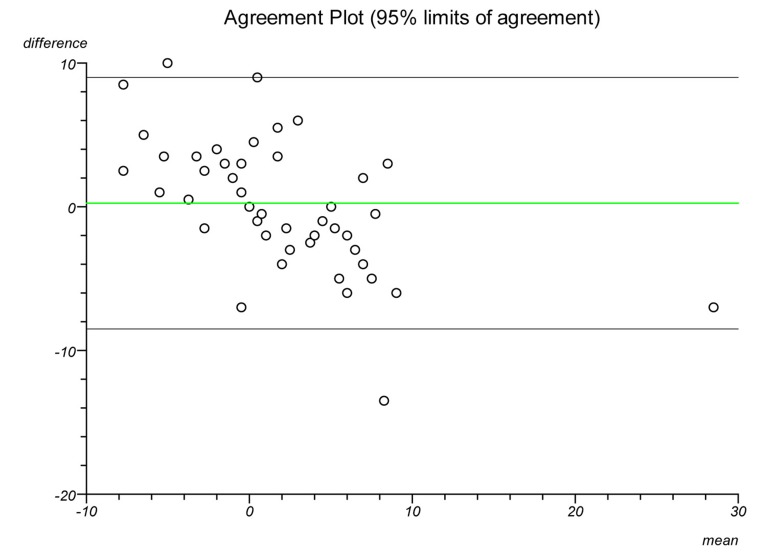
Radiographic plots using method 3 versus goniometer (extension)

**Figure 7:**
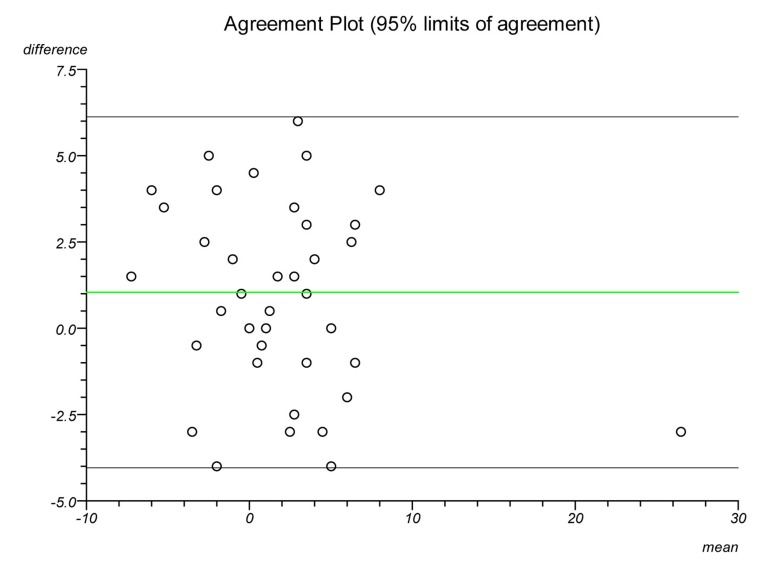
Radiographic plots using method 4 versus goniometer (extension)

**Figure 8:**
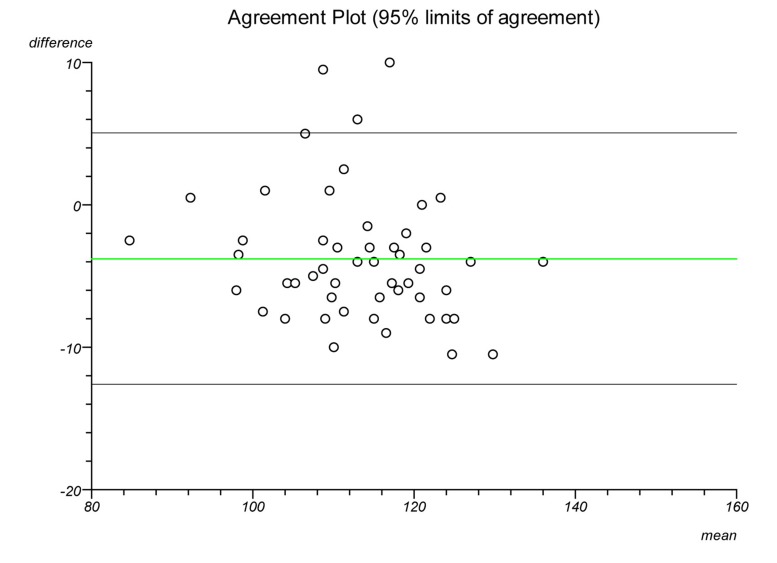
Radiographic plots using method 1 versus goniometer (flexion)

**Figure 9:**
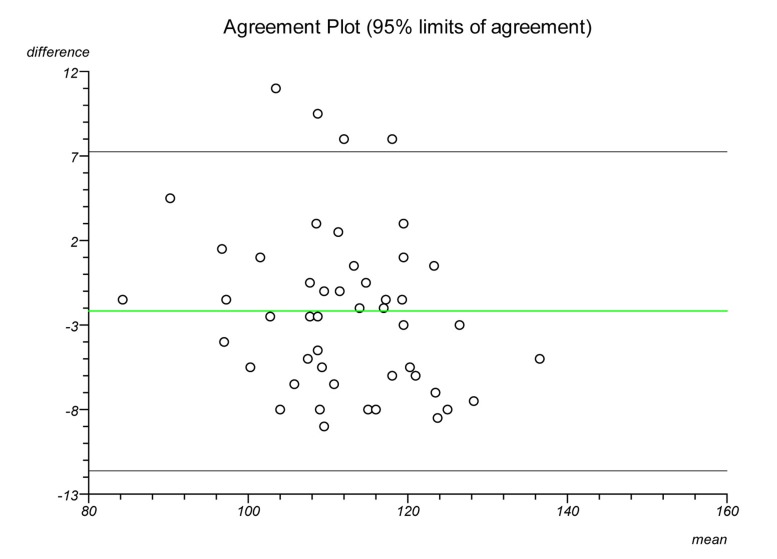
Radiographic plots using method 2 versus goniometer (flexion)

**Figure 10:**
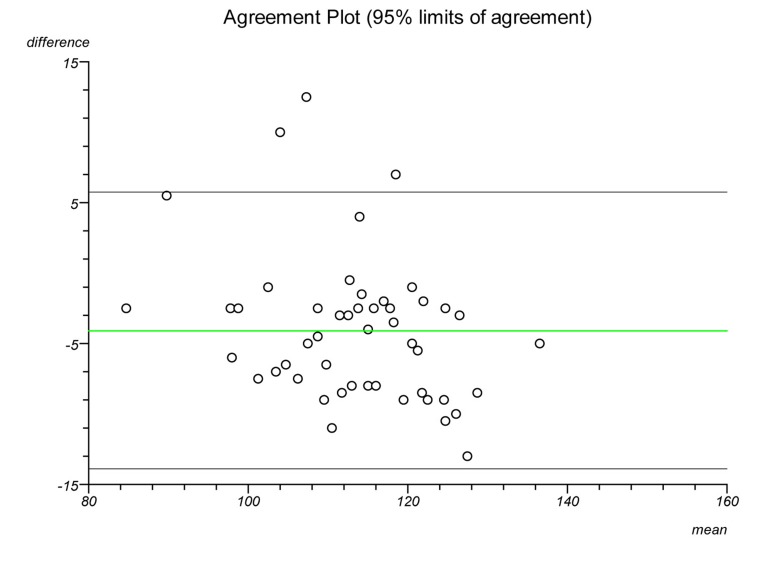
Radiographic plots using method 3 versus goniometer (flexion)

**Figure 11:**
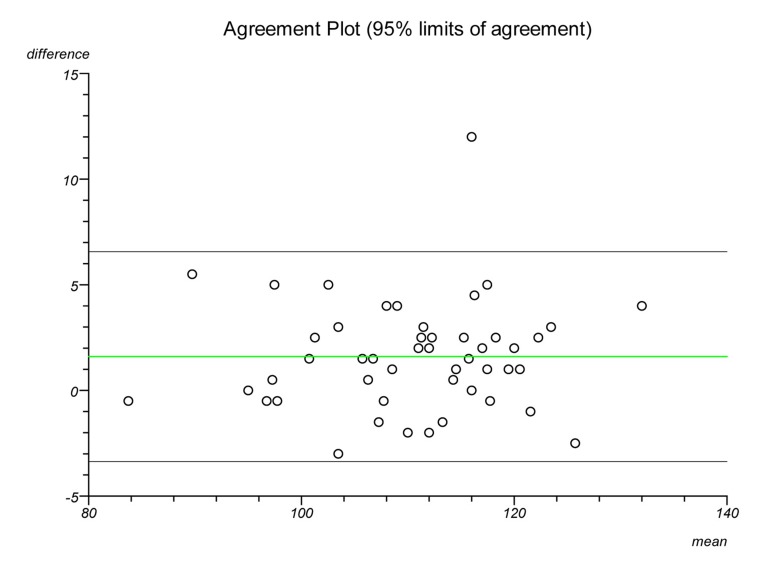
Radiographic plots using method 4 versus goniometer (flexion)

The Bland–Altman graphs (Figs 4–11) plot the mean of the paired measurements (x-axis) against their difference (y-axis). The mean of all the differences is shown by the green line. There is a trend, in flexion and extension, for the difference in measurements between goniometer and radiograph to get larger and more negative. This does not appear to be the case for method 4.

## Discussion

The results of this study show that range of motion of the knee in full active flexion and extension can be measured accurately using radiographs. The most accurate technique is method 4, described above. This allows us to assess post-arthroplasty range of motion of the knee on radiographs instead of in a consultation in the outpatient department.

Our unit performed 625 TKRs in 2009. We arrange follow-up appointments for routine post-operative care at six weeks from surgery then one, two, five and ten years. Routine radiographs are obtained at appointments after six weeks. Routine outcome measures currently in use in clinic and in the literature following TKRs include serial questionnaire derived scores, radiographs and evaluation of range of motion of the knee.^16^ Our 625 patients would generate 3,125 outpatient episodes over 10 years. The virtual clinic system would reduce the outpatient burden by 2,437 patient episodes, assuming 10% of patients may require a second visit at some point over the 10 years.

In 2009–2010 we generated 4,155 elective outpatient follow-up visits, costing £344,865. If our ratio for new to follow-up patients had been in the upper quartile range of 1:1.3,[ref] the cost to the commissioners (NHS Devon) would have been £191,398, representing a saving of £153,467. This is a 45% reduction in costs and would enable NHS Devon to provide evidence of compliance with the Quality, Innovation, Productivity and Prevention agenda.^17^ The disadvantage is that the RD&E would lose that revenue from NHS Devon but the clinic capacity could be reused for new patients.

The catchment area for the RD&E is large and mostly rural, covering 1,000 square miles. By using serial postal questionnaires and radiographic analysis of the range of motion of a knee post-operatively, we hope to be able to reduce the inconvenience to patients having to travel to the RD&E, in keeping with delivering services more locally as recommended in *The NHS Plan* and *Our Health, Our Care, Our Say*.^18,19^ We would also reduce our ratio for new to follow-up patients in outpatient clinics and, in turn, increase new patient capacity.

The knee surgeons at the Exeter Knee Reconstruction Unit use the Scorpio® total knee replacement almost exclusively. This prosthesis has performed well over the medium term,^16,20^ with survivorship of 99.3% up to nine years. No longer term data exist in the orthopaedic literature so collection of our own data in this way will provide us with an excellent means of assessing our outcomes. These are essential data that patients need to know for fully informed consent.

Range of motion is a major component of some knee scoring systems^21^ but, more importantly, from a patient’s perspective, range of motion relates directly to the function of the knee. It is associated inherently with implant positioning. If alignment of the prosthesis is incorrect, this can lead to abnormal wear,^22,23^ premature loosening,^24–26^ and patellofemoral problems.^28–31^ Routine radiographs include a standing anteroposterior view and lateral view as essential and, to correctly evaluate radiographic outcome, a long-leg alignment and skyline view are necessary. We propose that this standard could be changed to reflect not only assessment of loosening but incorporating range of motion into the series as a surrogate for function when combined with subjective outcome data. Our subjective outcome data would include serial validated questionnaires (postal or internet-based) to complement the radiographic surveillance.

## Conclusions

Many ways to reduce costs in the outpatient department have been published. Carter *et al* have shown that specialist nurse practitioners can reduce the cost of follow-up appoitnments to a department.^32^ Wasson *et al* showed that substituting some follow-up visits with a telephone conversation was advantageous, resulting in less medication use, fewer admissions (both planned and unplanned) and shorter hospital stays.^33^ We aim to reduce our outpatient costs and improve our ratio for new to follow-up patients in line with Department of Health guidelines by using virtual clinics. We have shown that measuring range of motion of the knee after arthroplasty on a suitable radiograph has high reliability and accuracy.
